# Supplementary figures and images for: Green synthesis of silver nanoparticles from Brownlowia tersa leaf extract: multifaceted evaluation of antibacterial, antioxidant, cytotoxic, and anti-alzheimer potential
Source: PLoS One. 2025 Nov 4;20(11):e0335524. doi: 10.1371/journal.pone.0335524 (PMC12585095; doi:10.1371/journal.pone.0335524)

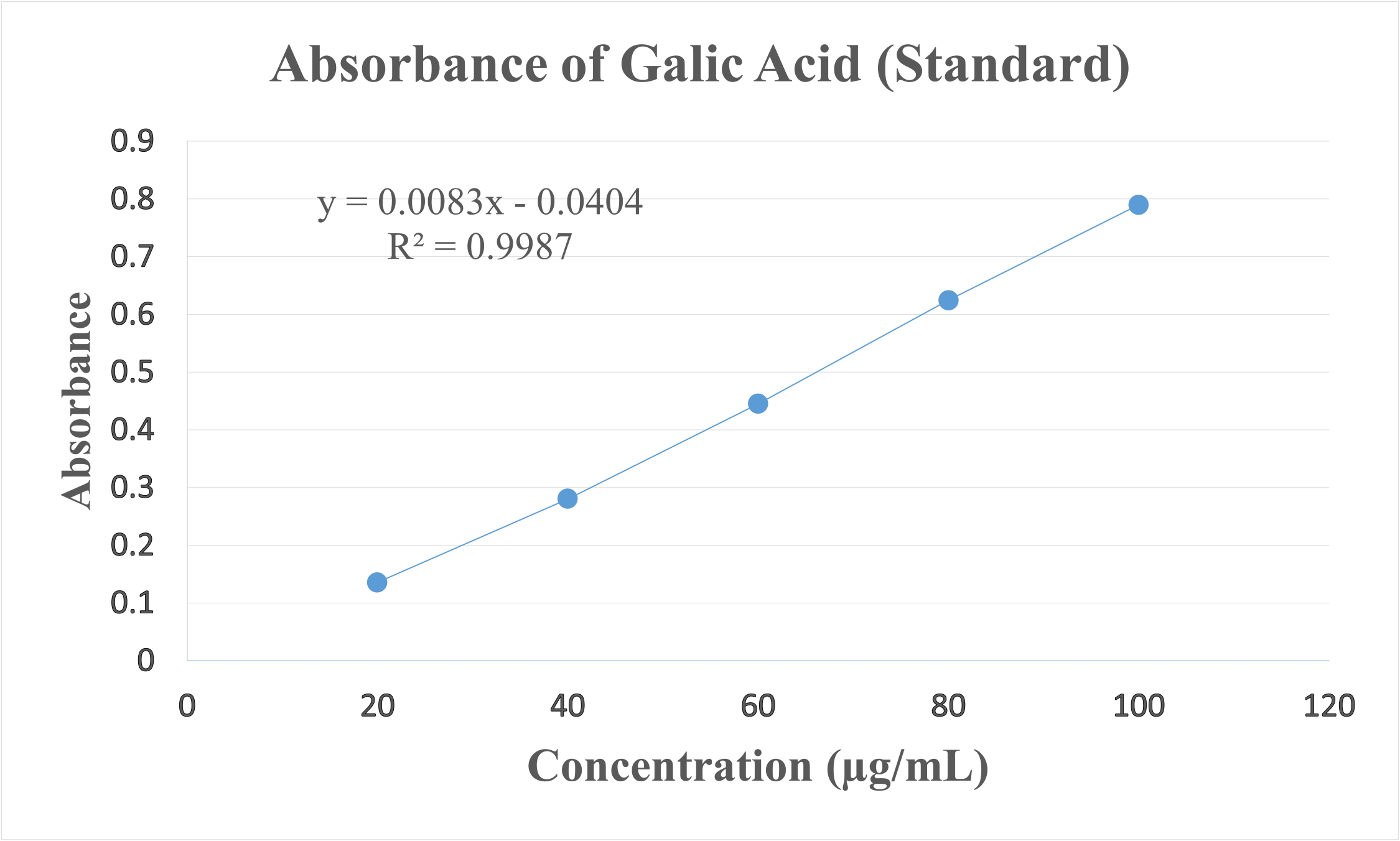

Supplement: S1 Fig — (TIF) [file pone.0335524.s001.tif]

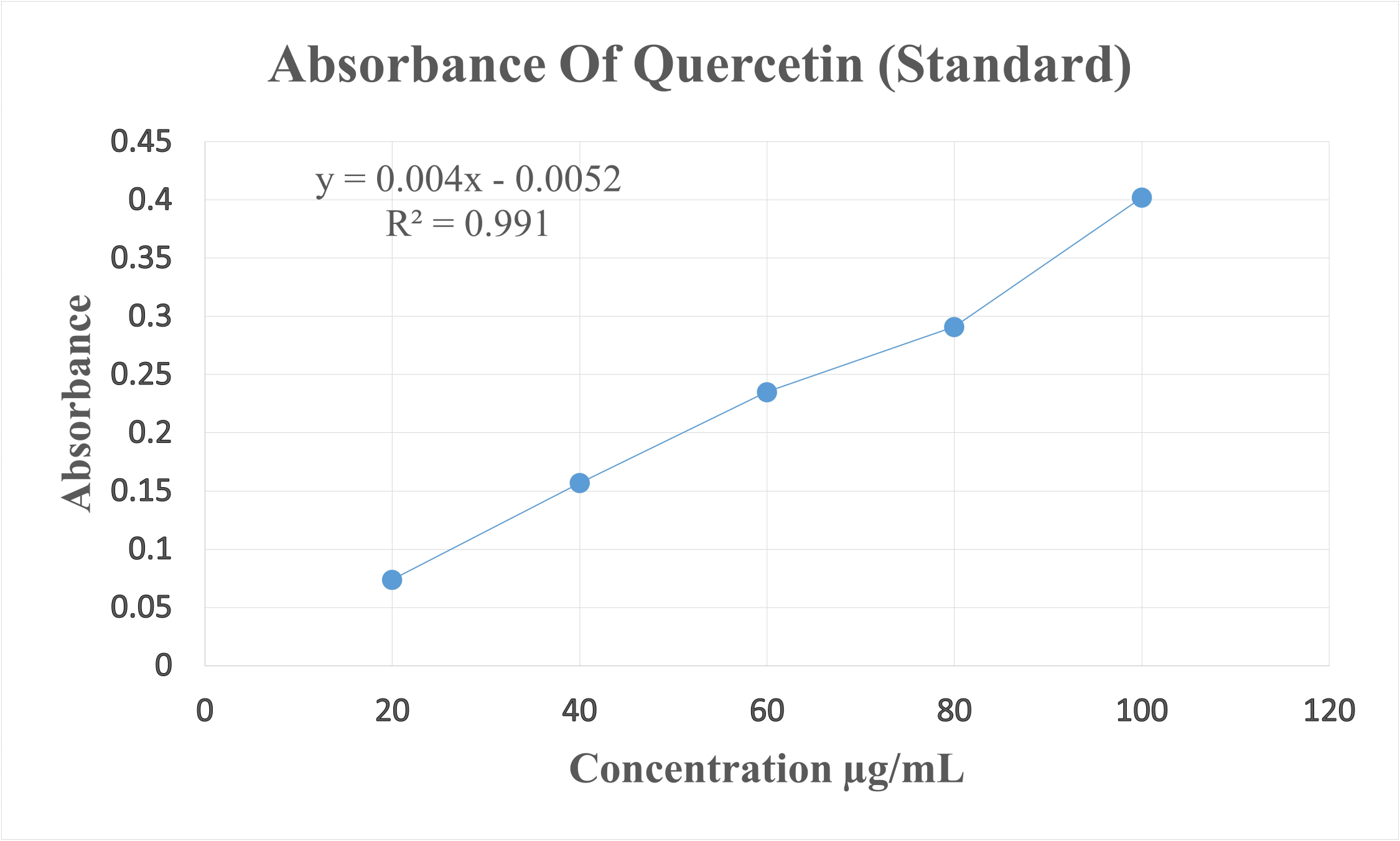

Supplement: S2 Fig — (TIF) [file pone.0335524.s002.tif]

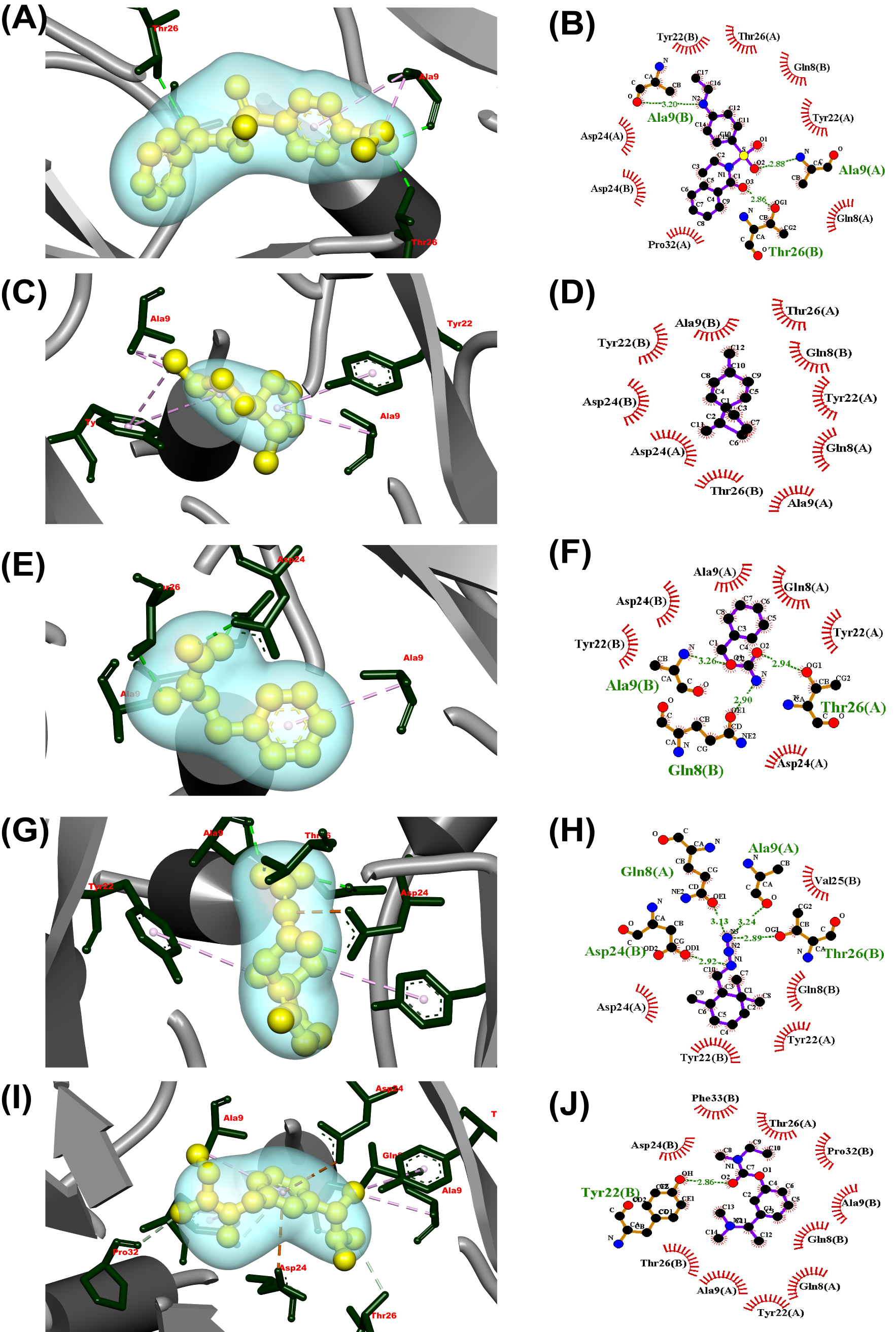

Supplement: S3 Fig — On one side, we have the three-dimensional complex of protein-ligand interaction, and on the other, we have the two-dimensional complex. A. Benzamide, N-ethyl-N-[(4-ethylaminophenyl) sulfonyl]- Amyloid A4 protein (PDB ID: 1AAP) B. trans, trans-1,8-Dimethylspiro [4.5] decane – Amyloid A4 protein (PDB ID: 1AAP) C. BENZYLCARBAMAT- Amyloid A4 protein (PDB ID: 1AAP) D. 2-Azidomethyl-1,3,3-trimethyl-cyclohexene - Amyloid A4 protein (PDB ID: 1AAP) E. Rivastigmine- Amyloid A4 protein (PDB ID: 1AAP). (TIF) [file pone.0335524.s003.tif]

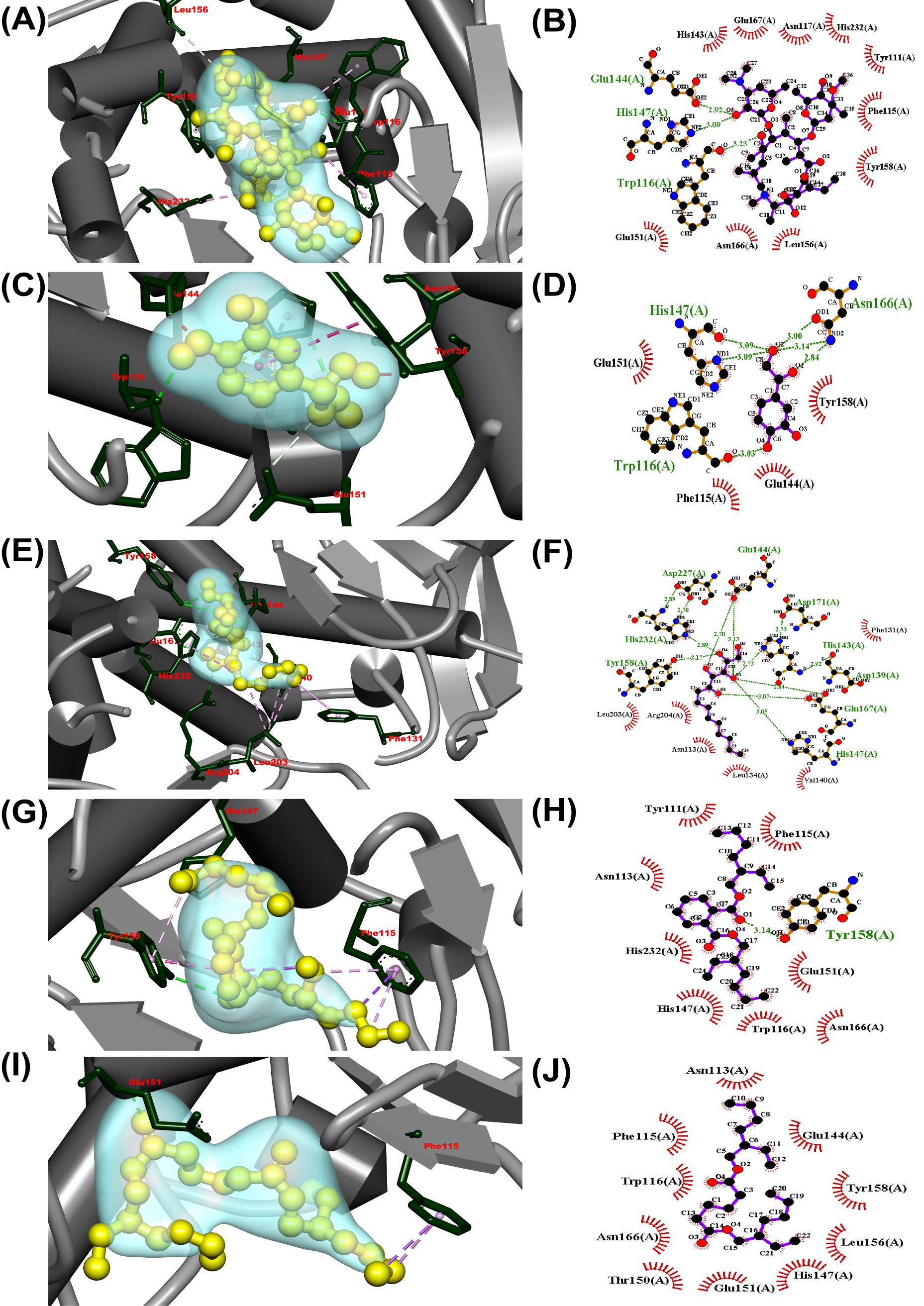

Supplement: S4 Fig — On one side, we have the three-dimensional complex of protein-ligand interaction, and on the other, we have the two-dimensional complex. A. Azithromycin- Tyrosyl-tRNA synthetase (PDB ID: 1JIJ) B. 3,4-Dihydroxyphenylglycol- Tyrosyl-tRNA synthetase (PDB ID: 1JIJ) C. Bis(2-ethylhexyl) phthalate- Tyrosyl-tRNA synthetase (PDB ID: 1JIJ) D. D-Mannotetradecane-1,2,3,4,5-pentaol - Tyrosyl-tRNA synthetase (PDB ID: 1JIJ) E. Hexanedioic acid, bis(2-ethylhexyl) ester – Tyrosyl-tRNA synthetase (PDB ID: 1JIJ). (TIF) [file pone.0335524.s004.tif]

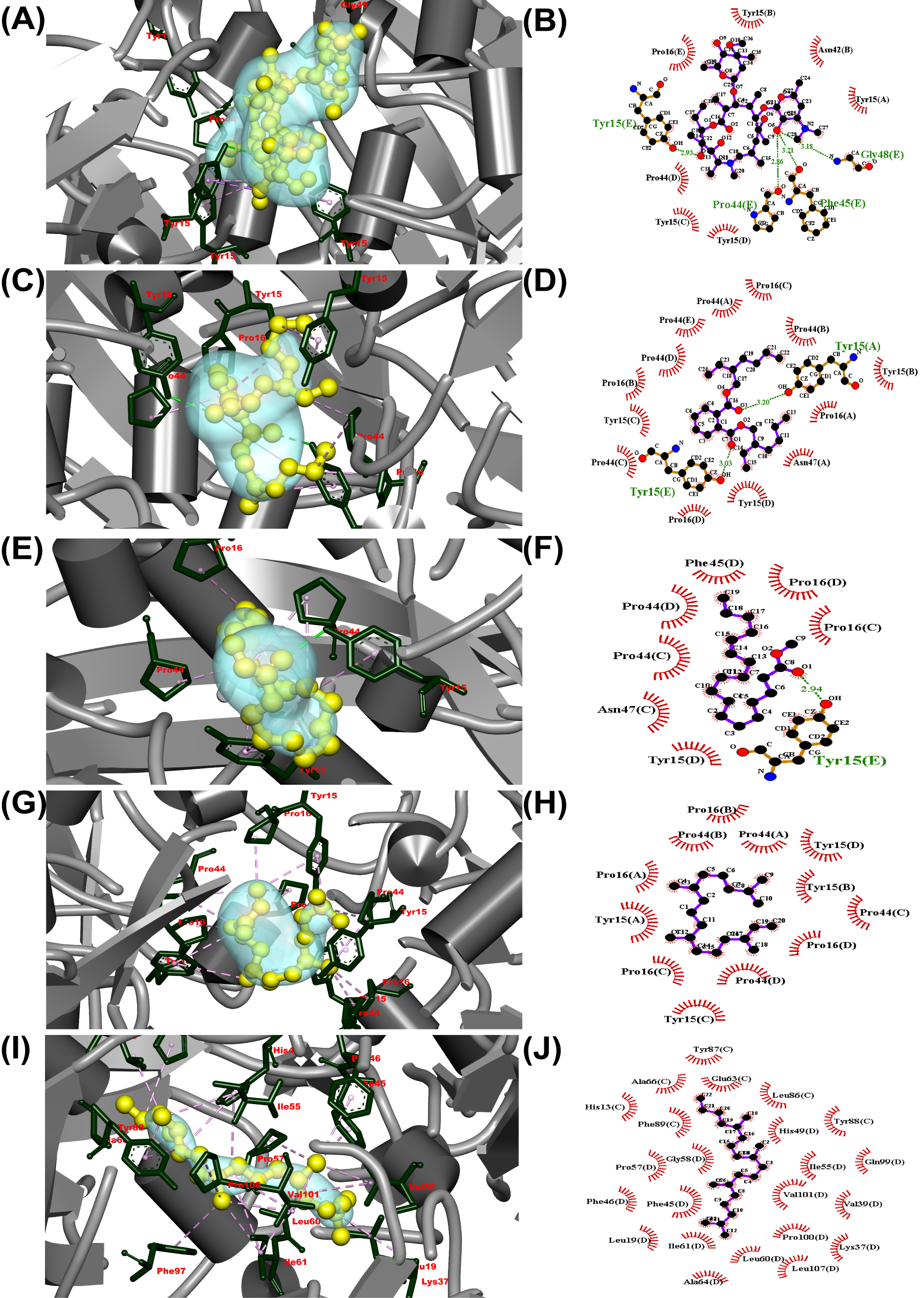

Supplement: S5 Fig — On one side, we have the three-dimensional complex of protein-ligand interaction, and on the other, we have the two-dimensional complex. A. Azithromycin- (3R)-hydroxymyristoyl-[acyl carrier protein] dehydratase (PDB ID: 1U1Z) B. Bis(2-ethylhexyl) phthalate- (3R)-hydroxymyristoyl-[acyl carrier protein] dehydratase (PDB ID: 1U1Z) C. 13-Octadecenoic acid, methyl ester-(3R)-hydroxymyristoyl-[acyl carrier protein] dehydratase (PDB ID: 1U1Z) D. Neophytadiene- (3R)-hydroxymyristoyl-[acyl carrier protein] dehydratase (PDB ID: 1U1Z) E. OCTADECANE, 2,6,10,14-TETRAMETHYL- (3R)-hydroxymyristoyl-[acyl carrier protein] dehydratase (PDB ID: 1U1Z). (TIF) [file pone.0335524.s005.tif]

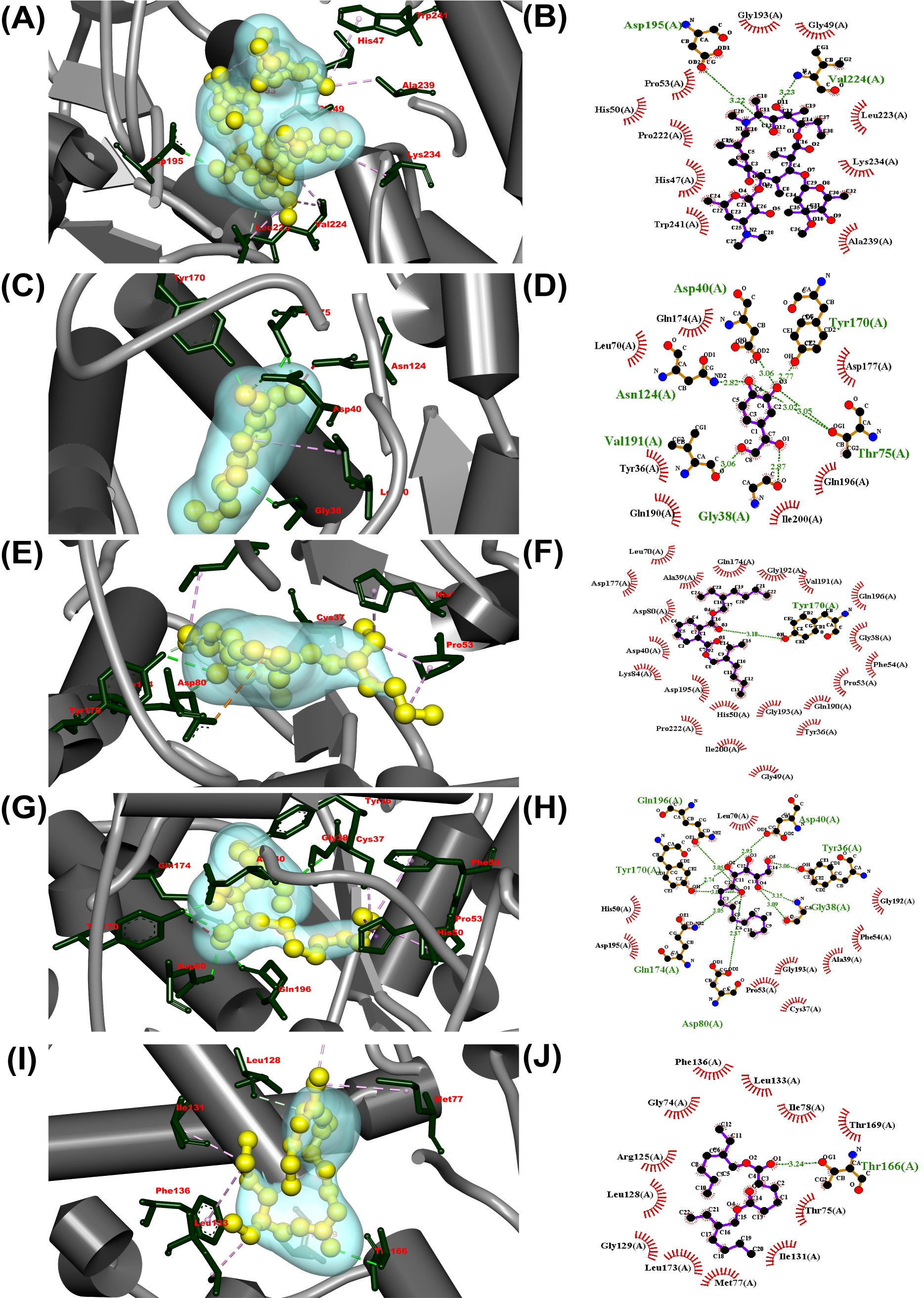

Supplement: S6 Fig — On one side, we have the three-dimensional complex of protein-ligand interaction, and on the other, we have the two-dimensional complex. A. Azithromycin- M4 metalloprotease protein (PDB ID: 1NPC) B. 3,4-Dihydroxyphenylglycol- M4 metalloprotease protein (PDB ID: 1NPC) C. D-Mannotetradecane-1,2,3,4,5-pentaol-M4 metalloprotease protein (PDB ID: 1NPC) D. Bis(2-ethylhexyl) phthalate- M4 metalloprotease protein (PDB ID: 1NPC) E. Hexanedioic acid, bis(2-ethylhexyl) ester- M4 metalloprotease protein (PDB ID: 1NPC). (TIF) [file pone.0335524.s006.tif]
