# Supplementary material for: Green synthesis of silver nanoparticles from Brownlowia tersa leaf extract: multifaceted evaluation of antibacterial, antioxidant, cytotoxic, and anti-alzheimer potential
Source: PLoS One. 2025 Nov 4;20(11):e0335524. doi: 10.1371/journal.pone.0335524 (PMC12585095; doi:10.1371/journal.pone.0335524)
Supplement: Attachment — Submitted filename: Response to Reviewers.docx [file pone.0335524.s009.docx]

22^nd^ June 2025

To

Emily Chenette

Editor-in-Chief,

PLOS One,

Dear Editor,

Special gratitude to you for allowing us to resubmit a revised version of our manuscript entitled as **“Green Synthesis of Silver Nanoparticles from Brownlowia tersa Leaf Extract: Multifaceted Evaluation of Antibacterial, Antioxidant, Cytotoxic, and Anti-Alzheimer Potential”**, (Manuscript ID: PONE-D-25-15530) to the PLOS One as a research article. We also appreciate the time and efforts of you and your reviewers have given for this meaningful feedback, which we believe has greatly helped us to enhance the clarity of our article significantly. We have made changes based on your and the reviewers' suggestions, and the revised sections are highlighted in yellow color throughout the revised manuscript. We hope that our response, which is included below, will deal successfully with each reviewer's specific points. Please let us know if you have any additional questions and comments.

Therefore, we want to express our special gratitude to you for your consideration of this manuscript.

**Sincerely Yours,**

Md. Nazmul Hasan, PhD,

Professor,

Department of Genetic Engineering and Biotechnology,

Jashore University of Science and Technology,

Jashore-7408, Bangladesh.

E-mail: [mn.hasan@just.edu.bd](mailto:mn.hasan@just.edu.bd)

Google Scholar Profile: <https://scholar.google.com/citations?user=SP1f9JEAAAAJ&hl=en>

ResearchGate Profile: <https://www.researchgate.net/profile/Dr-Md-Nazmul-Hasan>

ORCID ID: <https://orcid.org/0000-0002-9122-1129>

**Responses of Editor Comments**

**Comments of Editor 1:**

Comment 1). Please ensure that your manuscript meets PLOS ONE's style requirements, including those for file naming.

**>>Response:** The manuscript and all associated files have been revised to comply with PLOS ONE’s style requirements, including proper file naming conventions

**Comments of Editor 1:**

Comment 2). PLOS requires an ORCID iD for the corresponding author in Editorial Manager on papers submitted after December 6th, 2016. Please ensure that you have an ORCID iD and that it is validated in Editorial Manager. To do this, go to ‘Update my Information’ (in the upper left-hand corner of the main menu), and click on the Fetch/Validate link next to the ORCID field. This will take you to the ORCID site and allow you to create a new iD or authenticate a pre-existing iD in Editorial Manager.

**>>Response:** I will complete the creation and validation of my ORCID iD in Editorial Manager following the provided instructions.

**Comments of Editor 1:**

Comment 3). To comply with PLOS ONE submissions requirements, in your Methods section, please provide additional information regarding the experiments involving animals and ensure you have included details on (1) methods of sacrifice, (2) methods of anesthesia and/or analgesia, and (3) efforts to alleviate suffering.

**>>Response:** Thank you for the feedback. We have revised the *Experimental Animals* subsection in the Methods section to include detailed information on (1) methods of anesthesia and analgesia, (2) method of sacrifice, and (3) efforts to alleviate animal suffering, in compliance with PLOS ONE’s animal research reporting requirements.

**Comments of Editor 1:**

Comment 4). Your ethics statement should only appear in the Methods section of your manuscript. If your ethics statement is written in any section besides the Methods, please delete it from any other section.

**>>Response:** The ethics statement has been retained only in the Methods section and removed from all other parts of the manuscript, in accordance with PLOS ONE guidelines.

**Comments of Editor 1:**

Comment 5). Please remove your figures from within your manuscript file, leaving only the individual TIFF/EPS image files, uploaded separately. These will be automatically included in the reviewers’ PDF.

**>>Response:** All figures have been removed from the manuscript file and uploaded separately as individual TIFF/EPS files, as per PLOS ONE submission guidelines

**Comments of Editor 1:**

Comment 6). Please ensure that your manuscript meets PLOS ONE's style requirements, including those for file naming.

**>>Response:** The manuscript and all associated files have been revised to comply with PLOS ONE’s style requirements, including proper file naming conventions

**Additional Comments of Editor:**

**Comments of Editor 1:**

Comment 1). Is the manuscript technically sound, and do the data support the conclusions?

The manuscript must describe a technically sound piece of scientific research with data that supports the conclusions. Experiments must have been conducted rigorously, with appropriate controls, replication, and sample sizes. The conclusions must be drawn appropriately based on the data presented.

**>>Response:** We confirm that the manuscript presents a technically sound study. All experiments were conducted rigorously using appropriate controls, sufficient replication, and adequate sample sizes. The data have been thoroughly analyzed, and the conclusions are directly supported by the results presented. We have reviewed the manuscript to ensure that all interpretations align accurately with the data, and have clarified relevant sections where necessary to reinforce this alignment

**Comments of Editor 1:**

Comment 2). Has the statistical analysis been performed appropriately and rigorously?

**>>Response:** Statistical analyses were performed appropriately using validated methods, with details provided in the Methods section. Results include significance values and proper controls to ensure rigor

**Comments of Editor 1:**

Comment 3). Have the authors made all data underlying the findings in their manuscript fully available?

The PLOS Data policy requires authors to make all data underlying the findings described in their manuscript fully available without restriction, with rare exception (please refer to the Data Availability Statement in the manuscript PDF file). The data should be provided as part of the manuscript or its supporting information, or deposited to a public repository. For example, in addition to summary statistics, the data points behind means, medians and variance measures should be available. If there are restrictions on publicly sharing data—e.g. participant privacy or use of data from a third party—those must be specified.

**>>Response:** All data underlying the findings are fully available as described in the Data Availability Statement. Where applicable, data have been included in the manuscript, supporting information. Any restrictions are clearly specified in accordance with PLOS policy

**Comments of Editor 1:**

Comment 4). Is the manuscript presented in an intelligible fashion and written in standard English?

PLOS ONE does not copyedit accepted manuscripts, so the language in submitted articles must be clear, correct, and unambiguous. Any typographical or grammatical errors should be corrected at revision, so please note any specific errors here.

**>>Response:** The manuscript has been carefully reviewed and revised to ensure clarity, correctness, and standard English usage. All typographical and grammatical errors have been addressed

**Comments of Editor 1:**

Comment 5). Please use the space provided to explain your answers to the questions above. You may also include additional comments for the author, including concerns about dual publication, research ethics, or publication ethics. (Please upload your review as an attachment if it exceeds 20,000 characters)

**>>Response:** Thank you for your thorough review and constructive comments. We have carefully addressed each point raised to improve the manuscript’s quality and compliance with PLOS ONE policies. We confirm that there are no concerns regarding dual publication, and all research and publication ethics have been fully observed.

**Responses of Reviewer Comments**

**Comments of Reviewer 1:**

Comment 1). On page 10, line 222, authors should revise first sentence to make it sensible!

**>>Response:** I changed it.

Comment 2). On page 13, line 286, how the 80 mice were divided into four groups having 5 mice in each group?

**>>Response:** Thank you for pointing this out. There appears to be a discrepancy in the group allocation. The 80 mice should have been divided into 16 groups, with each group containing 5 mice, not 4 groups as originally stated. We have revised the manuscript to reflect the correct division of the mice into 16 groups, each consisting of 5 mice, for the in vivo experiments.

Comment 3). A large number of references are used in this manuscript which is not as norms of “original manuscript” writing! References should be updated with recent ones only and limit up to 50 or less!

**>>Response:** In accordance with the reviewer’s guidance, we have updated and streamlined our reference list. Less relevant and outdated citations have been removed, bringing the total number of references to 50, in line with the journal’s standards for original research articles

Comment 4) Table 1 show very unusual results regarding phenolic contents; need for counter check or valid discussion should be incorporated with reported evidences.

**>>Response:** Thank you for your valuable feedback regarding the phenolic content data presented in Table 1. In response to your observation of unusual results, I have re-conducted the experiment with the same methodology to ensure the accuracy and consistency of the measurements.

The updated results for phenolic content are as follows:

B. tersa: 422.63 ± 2.90 mg gallic acid equivalent/g sample

B. tersa leaves AgNPs: 48.29 ± 2.73 mg gallic acid equivalent/g sample

These values show a slight decrease in the mean compared to the initial results (442.63 ± 25.40 for B. tersa and 50.29 ± 5.73 for B. tersa leaves AgNPs). Importantly, the standard deviation has been significantly reduced, indicating improved consistency and precision in the re-test. I also performed a one-sample t-test to assess the statistical significance, and the data showed no significant difference from the hypothesized values, further supporting the validity of the results. This re-testing has addressed the concerns regarding the variability in the original measurements, and the updated data now provide more reliable and reproducible results.

Comment 5). Antioxidant activity clearly indicated negative results of synthesized nanoparticles which are not properly interpreted in results!

**>>Response:** I interpret properly according to your instructions.

Comment 6). Antibacterial activity is also not remarkable. Although it shows almost same zone of inhibition against 2 selected bacteria as that of standard but why not use advance available antibiotic as standard? Moreover, the synthesized nano formations not show any improved results in increasing concentrations!

**>>Response:** Thank you for your comment. In this study, no advanced antibiotics were used as standards, as the focus was on evaluating the antibacterial potential of Brownlowia tersa leaf-derived silver nanoparticles (AgNPs). While the AgNPs showed a similar zone of inhibition against Staphylococcus aureus and Pseudomonas aeruginosa as the standard, the lack of dose-dependent improvement may be due to nanoparticle aggregation at higher concentrations, which reduces their effective surface area. Future studies will explore the use of advanced antibiotics as standards, optimize concentrations, and test a broader range of bacterial strains to better assess the AgNPs' antibacterial potential.

Comment 7). The authors aim to demonstrate Green Synthesis of Silver Nanoparticles from Brownlowia tersa Leaf Extract and their Multifaceted Evaluation of Antibacterial, Antioxidant, Cytotoxic, and Anti-Alzheimer Potential however, the data does not fully support the conclusion

**>>Response:** The discussion and conclusion have been refined to better align the data with the claims, providing a more balanced interpretation of the Green Synthesis of Silver Nanoparticles and their antibacterial, antioxidant, cytotoxic, and anti-Alzheimer potentials.

Comment 8). While the study appears to be sound, the language is unclear, making it difficult to follow. I advise the authors work with a writing coach or copyeditor to improve the flow and readability of the text. further, please arrange manuscript as per standard, usually research articles should fit within the range of 6 000 to 12 000 words, including footnotes

**>>Response:** Thank you for your suggestion. The manuscript has been reviewed, and I confirm that the total word count for the main text (from Abstract to Conclusion) falls within the recommended 6,000-to-12,000-word range as per PLOS ONE guidelines.

Comments 9). How the results of elevated plus test are significantly higher as mentioned by authors! Wrong interpretation and unsatisfactory discussion are done regarding this activity.

**>>Response:** Thank you for your valuable feedback. Upon reviewing the data and your comments, I identified and corrected the statistical analysis related to the Elevated Plus Maze test. The previous interpretation inaccurately reported the significance of the results. After re-analysis using the appropriate statistical methods, the data now accurately reflect the behavioral outcomes, and the discussion has been updated accordingly to ensure a more precise and scientifically valid interpretation of the EPM activity

**Responses of Reviewer Comments**

**Comments of Reviewer 2:**

Comment 1). Abstract lacks quantitative key results and any study limitations. It is recommended to add statistical data like specific antibacterial or antioxidant activity values. Also add a sentence on current challenges of the approaches to strengthen impact.

**>>Response:** I changed it according to the instructions.

Comment 2). Introduction lack the rationale of focused discussion on Brownlowia tersa for their study. Give strong justification of the selection of B. tersa as a reducing agent.

**>>Response:** Thank you for highlighting this important point. I have revised the Introduction to provide a clearer rationale for focusing on Brownlowia tersa as the reducing agent. The updated section now includes a strong justification based on its phytochemical profile, traditional medicinal relevance, and potential for bio-reductive activity, supporting its suitability for this study.

Comment 3). Characterization methods such as UV-vis, FESEM and FTIR lack sufficient details. Provide detailed protocols for all assays and characterization methods.

**>>Response:** Thank you for your helpful comment. I have revised the manuscript to include detailed protocols for all characterization methods, including UV–Vis spectroscopy, FESEM, and FTIR analysis. These sections now provide sufficient methodological information to ensure clarity and reproducibility.

Comment 4). The manuscript lacks detailed protocols like controls, replicates, statistical analysis methods in biological assays. It is recommended to add information about controls, replicates, and statistical analysis. Also explicitly explain computational methodology in depth for reproducibility.

**>>Response:** Thank you for pointing this out. I have revised the manuscript to include detailed descriptions of the biological assay protocols, including information on controls, number of replicates, and the statistical analysis methods used. Additionally, the computational methodology section has been expanded to ensure clarity and reproducibility

Comment 5). Results and Discussion lack comparison with pre-existing literature. Add comparative studies of statistical data with previous studies. Elaborate discussions on mechanistic analysis of findings.

**>>Response:** I did it according to your instructions.

Comment 6). The manuscript lacks potential barriers like large-scale production, monitoring or toxicity. It is suggested to discuss these barriers for real-world application.

**>>Response:** In conclusion, potential real-world barriers, including challenges in large-scale production, monitoring, and toxicity, have been addressed to highlight practical considerations for future application.

Comment 7). The conclusion lacks future research perspectives or broader implication. It is recommended to add future direction, such as in vivo validation or formulation studies. Also add a sentence that emphasize the need of safety and regulatory evaluation.

**>>Response:** The conclusion section has been expanded to include future research perspectives, such as in vivo validation, formulation studies, and the essential need for safety and regulatory evaluations.
