# Supplementary material for: Green synthesis of silver nanoparticles from Brownlowia tersa leaf extract: multifaceted evaluation of antibacterial, antioxidant, cytotoxic, and anti-alzheimer potential
Source: PLoS One. 2025 Nov 4;20(11):e0335524. doi: 10.1371/journal.pone.0335524 (PMC12585095; doi:10.1371/journal.pone.0335524)
Supplement: Attachment — Submitted filename: Response_to_Reviewers_auresp_2.docx [file pone.0335524.s010.docx]

26^th^ July 2025

To

Emily Chenette

Editor-in-Chief,

PLOS One,

Dear Editor,

Special gratitude to you for allowing us to resubmit a revised version of our manuscript entitled as **“Green Synthesis of Silver Nanoparticles from Brownlowia tersa Leaf Extract: Multifaceted Evaluation of Antibacterial, Antioxidant, Cytotoxic, and Anti-Alzheimer Potential”**, (Manuscript ID: PONE-D-25-15530R1) to the PLOS One as a research article. We also appreciate the time and efforts of you and your reviewers have given for this meaningful feedback, which we believe has greatly helped us to enhance the clarity of our article significantly. We have made changes based on your and the reviewers' suggestions, and the revised sections are highlighted in yellow color throughout the revised manuscript. We hope that our response, which is included below, will deal successfully with each reviewer's specific points. Please let us know if you have any additional questions and comments.

Therefore, we want to express our special gratitude to you for your consideration of this manuscript.

**Sincerely Yours,**

Md. Nazmul Hasan, PhD,

Professor,

Department of Genetic Engineering and Biotechnology,

Jashore University of Science and Technology,

Jashore-7408, Bangladesh.

E-mail: [mn.hasan@just.edu.bd](mailto:mn.hasan@just.edu.bd)

Google Scholar Profile: <https://scholar.google.com/citations?user=SP1f9JEAAAAJ&hl=en>

ResearchGate Profile: <https://www.researchgate.net/profile/Dr-Md-Nazmul-Hasan>

ORCID ID: <https://orcid.org/0000-0002-9122-1129>

**Responses of Editor Comments**

**Comments of Editor 1:**

Comment 1).  If the authors have adequately addressed your comments raised in a previous round of review and you feel that this manuscript is now acceptable for publication, you may indicate that here to bypass the “Comments to the Author” section, enter your conflict-of-interest statement in the “Confidential to Editor” section, and submit your "Accept" recommendation

**>>Response:** Thank you for your review and for providing the opportunity to revise the manuscript. We have carefully addressed the reviewer comments in the revised manuscript, and we believe that all issues have been adequately resolved.

The manuscript has been updated with a more thorough interpretation of the results, especially regarding antioxidant activity and elevated plus maze section, and the necessary revisions have been made in the Abstract and Discussion sections as per the reviewers' suggestions.

We feel the manuscript is now suitable for publication, and we respectfully recommend it for acceptance.

**Comments of Editor 1:**

Comment 2). Is the manuscript technically sound, and do the data support the conclusions?

**>>Response:** Yes, the manuscript is technically sound, and the data support the conclusions. The experimental methods are clearly described, and the results, including antioxidant and antimicrobial activities, have been appropriately interpreted and are consistent with the conclusions.

**Comments of Editor 1:**

Comment 3). Has the statistical analysis been performed appropriately and rigorously?

**>>Response:** Yes, the statistical analysis has been performed appropriately and rigorously. We used standard statistical methods to analyze the data, ensuring the validity of the results. Statistical significance has been clearly indicated where applicable, and the analysis supports the conclusions drawn in the manuscript

**Comments of Editor 1:**

Comment 4). Have the authors made all data underlying the findings in their manuscript fully available?

**>>Response:** Yes, all data underlying the findings are fully available within the manuscript. We have included all relevant data within the text and figures to support the conclusions drawn in the study

**Comments of Editor 1:**

Comment 5). Is the manuscript presented in an intelligible fashion and written in standard English?

**>>Response:** Yes, the manuscript is presented in an intelligible manner and written in standard English. We have carefully revised the text to ensure clarity, coherence, and proper grammar throughout the manuscript.

**Comments of Editor 1:**

Comment 6). PLOS authors have the option to publish the peer review history of their article ([what does this mean?](https://track.editorialmanager.com/CL0/https:%2F%2Fjournals.plos.org%2Fplosone%2Fs%2Feditorial-and-peer-review-process%23loc-peer-review-history/1/010f019840dfc9b0-1ca1e3e2-1112-4aa9-b8e9-f080c61ffd95-000000/jVSagY9kzunZqTOL8DA24jaeuI039NYL-U2pXWKVpYU=221)). If published, this will include your full peer review and any attached files.


If you choose “no”, your identity will remain anonymous but your review may still be made public.

**>>Response:** Thank you for the clarification. We would prefer to keep our review anonymous and choose the option to not publish the peer review history. We understand that while our identity will remain anonymous, the review may still be made public

**Responses of Reviewer Comments**

**Comments of Reviewer 1:**

Comment 1). Revised manuscript is still lacking many corrections! Authors didn’t follow comments fully and now it must strictly revise before any further proceedings!!

**>>Response:** We apologize for any oversight in our previous revision. We have now thoroughly reviewed all the reviewer comments and have ensured that all points have been addressed properly. We have revised the manuscript to reflect the necessary changes and corrections, particularly focusing on areas that needed improvement.

**Comments of Reviewer 1:**

Comment 2). No proper corrections and discussion have been added regarding the “elevated plus test” as well as no improvements regarding antioxidant activity (in abstract especially). Both activities need proper and correct results interpretation!

**>>Response:** We appreciate the reviewer highlighting these areas. We have now included a more detailed discussion of the **elevated plus maze test** in the **Discussion** section, where we provide a more thorough interpretation of the results and their implications. Additionally, we have revised the **abstract** to include a clearer and more accurate interpretation of the **antioxidant activity** results. The differences in IC₅₀ values between AgNPs, plant extract, and ascorbic acid have been explained in more depth, and we’ve provided context for potential improvements in AgNP synthesis to enhance antioxidant capacity.

**Comments of Reviewer 1:**

Comment 3). Acknowledgement should be excluded or authors should acknowledge someone else other than themselves!

**>>Response:** We have revised the **Acknowledgements** section to exclude self-acknowledgment

**Responses of Reviewer Comments**

**Comments of Reviewer 2:**

Comment 1). No more comments, paper has been revised sufficiently so I recommend the publication of manuscript in current form.

**>>Response:** We sincerely thank the reviewer for their positive feedback. We are pleased to hear that the manuscript has been revised sufficiently. We appreciate your recommendation for publication and are grateful for your time and constructive suggestions throughout the review process
