# Supplementary material for: Green synthesis of silver nanoparticles from Brownlowia tersa leaf extract: multifaceted evaluation of antibacterial, antioxidant, cytotoxic, and anti-alzheimer potential
Source: PLoS One. 2025 Nov 4;20(11):e0335524. doi: 10.1371/journal.pone.0335524 (PMC12585095; doi:10.1371/journal.pone.0335524)
Supplement: Attachment — Submitted filename: Response_to_Reviewers_auresp_3.docx [file pone.0335524.s011.docx]

15^th^ August 2025

To

Emily Chenette

Editor-in-Chief,

PLOS One,

Dear Editor,

Special gratitude to you for allowing us to resubmit a revised version of our manuscript entitled as **“Green Synthesis of Silver Nanoparticles from Brownlowia tersa Leaf Extract: Multifaceted Evaluation of Antibacterial, Antioxidant, Cytotoxic, and Anti-Alzheimer Potential”**, (Manuscript ID: PONE-D-25-15530R2) to the PLOS One as a research article. We also appreciate the time and efforts of you and your reviewers have given for this meaningful feedback, which we believe has greatly helped us to enhance the clarity of our article significantly. We have made changes based on your and the reviewers' suggestions, and the revised sections are highlighted in yellow color throughout the revised manuscript. We hope that our response, which is included below, will deal successfully with each reviewer's specific points. Please let us know if you have any additional questions and comments.

Therefore, we want to express our special gratitude to you for your consideration of this manuscript.

**Sincerely Yours,**

Md. Nazmul Hasan, PhD,

Professor,

Department of Genetic Engineering and Biotechnology,

Jashore University of Science and Technology,

Jashore-7408, Bangladesh.

E-mail: [mn.hasan@just.edu.bd](mailto:mn.hasan@just.edu.bd)

Google Scholar Profile: <https://scholar.google.com/citations?user=SP1f9JEAAAAJ&hl=en>

ResearchGate Profile: <https://www.researchgate.net/profile/Dr-Md-Nazmul-Hasan>

ORCID ID: <https://orcid.org/0000-0002-9122-1129>

**Journal Requirements:**

**Comments 1**). If the reviewer comments include a recommendation to cite specific previously published works, please review and evaluate these publications to determine whether they are relevant and should be cited. There is no requirement to cite these works unless the editor has indicated otherwise. 

**>>Response:** The reviewer did not suggest any additional references, and after reviewing the manuscript, I believe that the references currently included are appropriate and sufficiently support the study's findings and discussion. I have not added any new references since the existing ones cover the relevant literature comprehensively

Comments 2). Please review your reference list to ensure that it is complete and correct. If you have cited papers that have been retracted, please include the rationale for doing so in the manuscript text, or remove these references and replace them with relevant current references. Any changes to the reference list should be mentioned in the rebuttal letter that accompanies your revised manuscript. If you need to cite a retracted article, indicate the article’s retracted status in the References list and also include a citation and full reference for the retraction notice.

**>>Response:** I have carefully reviewed the reference list to ensure that all citations are complete and correct. I can confirm that none of the cited papers have been retracted. If any retracted articles had been cited, I would have followed the guidelines by including the rationale in the manuscript or replaced them with relevant current references.

No changes were made to the reference list, as all citations are up-to-date and appropriate. If any modifications were made, they will be mentioned in the rebuttal letter accompanying the revised manuscript.

**Comment 3).**  Your ethics statement should only appear in the Methods section of your manuscript. If your ethics statement is written in any section besides the Methods, please delete it from any other section.

**>>Response:** We have revised the manuscript so that the ethics statement now appears only in the Methods section, as requested. It has been removed from all other sections.

**Responses of Editor Comments**

**Comments of Editor 1:**

Comment 1).  If the authors have adequately addressed your comments raised in a previous round of review and you feel that this manuscript is now acceptable for publication, you may indicate that here to bypass the “Comments to the Author” section, enter your conflict-of-interest statement in the “Confidential to Editor” section, and submit your "Accept" recommendation.

**>>Response:** The authors have successfully addressed all the comments raised in the previous round of review, and the manuscript is now in an acceptable form for publication.

**Comments of Editor 1:**

Comment 2). Is the manuscript technically sound, and do the data support the conclusions?
The manuscript must describe a technically sound piece of scientific research with data that supports the conclusions. Experiments must have been conducted rigorously, with appropriate controls, replication, and sample sizes. The conclusions must be drawn appropriately based on the data presented.

**>>Response:** Yes. The manuscript presents a technically sound piece of scientific research. The experiments were conducted with appropriate rigor, including proper controls, sufficient replication, and adequate sample sizes. The data presented in the manuscript clearly support the conclusions drawn by the authors. There are no apparent issues with the methodology or data analysis that would undermine the validity of the results.

**Comments of Editor 1:**

Comment 3). Has the statistical analysis been performed appropriately and rigorously?

**>>Response:** Yes, the statistical analysis has been performed appropriately and rigorously. We used standard statistical methods to analyze the data, ensuring the validity of the results. Statistical significance has been clearly indicated where applicable, and the analysis supports the conclusions drawn in the manuscript

**Comments of Editor 1:**

Comment 4). Have the authors made all data underlying the findings in their manuscript fully available?

The [PLOS Data policy](http://www.plosone.org/static/policies.action#sharing) requires authors to make all data underlying the findings described in their manuscript fully available without restriction, with rare exception (please refer to the Data Availability Statement in the manuscript PDF file). The data should be provided as part of the manuscript or its supporting information, or deposited to a public repository. For example, in addition to summary statistics, the data points behind means, medians and variance measures should be available. If there are restrictions on publicly sharing data—e.g. participant privacy or use of data from a third party—those must be specified.

**>>Response:** **Yes**. The authors have made all data underlying the findings fully available, as required by the PLOS Data policy. The data is included in the manuscript. There are no restrictions on publicly sharing the data

**Comments of Editor 1:**

Comment 5). Is the manuscript presented in an intelligible fashion and written in standard English?

PLOS ONE does not copyedit accepted manuscripts, so the language in submitted articles must be clear, correct, and unambiguous. Any typographical or grammatical errors should be corrected at revision, so please note any specific errors here.

**>>Response:** The manuscript is presented in an intelligible fashion and is written in standard English. I did not notice any major typographical or grammatical errors. The language is clear, correct, and unambiguous.

**Responses of Reviewer Comments**

**Comments of Reviewer 1:**

Comment 1). Please use the space provided to explain your answers to the questions above. You may also include additional comments for the author, including concerns about dual publication, research ethics, or publication ethics. (Please upload your review as an attachment if it exceeds 20,000 characters)

Reviewer #1: Although authors revised manuscript and now making some sense but still require to change conclusion accordingly!! please note that your formed NPs did not show any significant biological activity......so write conclusion regarding accurate findings!!

**>>Response:** Thank you for your valuable feedback. We have revised the conclusion to more accurately reflect the findings of our study. While the B. tersa-derived AgNPs demonstrated significant biological activity in antioxidant, and neuroprotective assays, we have clarified that no significant anxiolytic effects were observed in the Elevated Plus Maze test. We have also acknowledged the challenges associated with scaling up the synthesis process and the need for long-term monitoring of the AgNPs' stability, bioaccumulation, and potential toxicity.

We believe these changes provide a more accurate representation of our findings and the current limitations of the study. Future research directions, including in vivo validation and formulation studies, have been outlined to address these challenges and optimize the therapeutic potential of AgNPs.

**Comments of Reviewer 1:**

Comment 2). PLOS authors have the option to publish the peer review history of their article ([what does this mean?](https://journals.plos.org/plosone/s/editorial-and-peer-review-process#loc-peer-review-history)). If published, this will include your full peer review and any attached files.
If you choose “no”, your identity will remain anonymous but your review may still be made public.

**Do you want your identity to be public for this peer review?** For information about this choice, including consent withdrawal, please see our [Privacy Policy](https://www.plos.org/privacy-policy).

**>>Response:** I prefer to keep my identity anonymous for this peer review. I am comfortable with my review being made public, but I would like to remain anonymous in accordance with PLOS's policies.

Comment 3). If the authors have adequately addressed your comments raised in a previous round of review and you feel that this manuscript is now acceptable for publication, you may indicate that here to bypass the “Comments to the Author” section, enter your conflict-of-interest statement in the “Confidential to Editor” section, and submit your "Accept" recommendation.

**>>Response:** I have no conflict of interest regarding this manuscript
