# Supplementary material for: Green synthesis of silver nanoparticles from Brownlowia tersa leaf extract: multifaceted evaluation of antibacterial, antioxidant, cytotoxic, and anti-alzheimer potential
Source: PLoS One. 2025 Nov 4;20(11):e0335524. doi: 10.1371/journal.pone.0335524 (PMC12585095; doi:10.1371/journal.pone.0335524)
Supplement: Attachment — Submitted filename: Response_to_Reviewers_auresp_4.docx [file pone.0335524.s012.docx]

26^th^ September 2025

To

Emily Chenette

Editor-in-Chief,

PLOS One,

Dear Editor,

Special gratitude to you for allowing us to resubmit a revised version of our manuscript entitled as **“Green Synthesis of Silver Nanoparticles from Brownlowia tersa Leaf Extract: Multifaceted Evaluation of Antibacterial, Antioxidant, Cytotoxic, and Anti-Alzheimer Potential”**, (Manuscript ID: PONE-D-25-15530R3) to the PLOS One as a research article. We also appreciate the time and efforts of you and your reviewers have given for this meaningful feedback, which we believe has greatly helped us to enhance the clarity of our article significantly. We have made changes based on your and the reviewers' suggestions, and the revised sections are highlighted in yellow color throughout the revised manuscript. We hope that our response, which is included below, will deal successfully with each reviewer's specific points. Please let us know if you have any additional questions and comments.

Therefore, we want to express our special gratitude to you for your consideration of this manuscript.

**Sincerely Yours,**

Md. Nazmul Hasan, PhD,

Professor,

Department of Genetic Engineering and Biotechnology,

Jashore University of Science and Technology,

Jashore-7408, Bangladesh.

E-mail: [mn.hasan@just.edu.bd](mailto:mn.hasan@just.edu.bd)

Google Scholar Profile: <https://scholar.google.com/citations?user=SP1f9JEAAAAJ&hl=en>

ResearchGate Profile: <https://www.researchgate.net/profile/Dr-Md-Nazmul-Hasan>

ORCID ID: <https://orcid.org/0000-0002-9122-1129>

**Journal Requirements:**

**Comments 1**). If the reviewer comments include a recommendation to cite specific previously published works, please review and evaluate these publications to determine whether they are relevant and should be cited. There is no requirement to cite these works unless the editor has indicated otherwise. 

**>>Response:** The reviewer did not suggest any additional references, and after reviewing the manuscript, I believe that the references currently included are appropriate and sufficiently support the study's findings and discussion. I have not added any new references since the existing ones cover the relevant literature comprehensively

Comments 2). Please review your reference list to ensure that it is complete and correct. If you have cited papers that have been retracted, please include the rationale for doing so in the manuscript text, or remove these references and replace them with relevant current references. Any changes to the reference list should be mentioned in the rebuttal letter that accompanies your revised manuscript. If you need to cite a retracted article, indicate the article’s retracted status in the References list and also include a citation and full reference for the retraction notice.

**>>Response:** I have carefully reviewed the reference list to ensure that all citations are complete and correct. I can confirm that none of the cited papers have been retracted. If any retracted articles had been cited, I would have followed the guidelines by including the rationale in the manuscript or replaced them with relevant current references.

No changes were made to the reference list, as all citations are up-to-date and appropriate. If any modifications were made, they will be mentioned in the rebuttal letter accompanying the revised manuscript.

**Responses of Editor Comments**

**Comments of Editor 1:**

Comment 1).  Abstract lacks the details regarding the molecular docking and binding affinities.

**>>Response:** I have included molecular docking and binding affinity part.

**Comments of Editor 1:**

Comment 2). Line 127 & 128: degree Celsius, revolutions per minute: use standard abbreviation.

**>>Response:** Revised as suggested. “degree Celsius” has been changed to “°C” and “revolutions per minute” has been changed to “rpm.

**Comments of Editor 1:**

Comment 3). Line 148: 2.5.2 Anagesia: correct spelling.

**>>Response:** Corrected as suggested. “Anagesia” has been revised to “Analgesia

**Comments of Editor 1:**

Comment 4). Line 153: mention guideline ref

**>>Response:** Guideline reference has been included as per the provided instructions to ensure alignment with the specified standards

**Comments of Editor 1:**

Comment 5). Line 157 20g: 20 g,

**>>Response:** Corrected spacing in '20g' to '20 g' for consistency with formatting guideline

**Responses of Editor Comments**

**Comments of Editor 1:**

Comment 6).  Line 172: mention the change in spectra or the metal peak, both in discussion and spectra fig 2.

**>>Response:** I have written it.

**Comments of Editor 1:**

Comment 7). Line 18 hours

**>>Response:** Thank you for your feedback. The 96-well plate was kept in incubation for 18 hours at 37°C. We have ensured consistency in the manuscript by clearly specifying the incubation period throughout. Please let us know if further clarification is needed

**Comments of Editor 1:**

Comment 8). Line 288: sterile Nutrient

**>>Response:** Thank you for your feedback. 'Sterile nutrient' refers to autoclaved nutrient agar, and we will update the manuscript to specify this term for clarity and consistency

**Comments of Editor 1:**

Comment 9). Kindly mention and discuss the metal peaks in FT-IR Discussion and in Figure also fig 3.

**>>Response:** I have written it.

**Comments of Editor 1:**

Comment 10). In molecular docking section, mention the grid box and x,y, z coordinates details

**>>Response:** I have included it.

**Comments of Editor 1:**

Comment 11).  Use same abbreviation for NP as AgNP, in Fig 11

**>>Response:** Thank you for your suggestion. We will revise Figure 11 to use the same abbreviation 'AgNP' instead of 'NP' for consistency throughout the manuscript

**Comments of Editor 1:**

Comment 12). Justify the selection of doses for brine shrimp lethality assay and DPPH

**>>Response:** Thank you for your comment. The doses selected for the brine shrimp lethality assay and DPPH assay were based on preliminary studies and literature references that indicated their efficacy in evaluating the toxicity and antioxidant potential of the synthesized nanoparticles. For the brine shrimp lethality assay, we chose a range of doses that would allow us to assess the toxicity at different concentrations, ensuring we captured a broad spectrum of potential effects. Similarly, for the DPPH assay, the doses were selected to include both low and high concentrations, allowing for a comprehensive assessment of antioxidant activity across a range of concentrations, consistent with standard protocols used in similar studies.

**Comments of Editor 1:**

Comment 13). Fig 4: need much clear picture as evidence for size reduction.

**>>Response:** I have increased resolution of this figure

**Comments of Editor 1:**

Comment 14). Figure 5 IC 50: rectify it as IC 50

**>>Response:** I have done it.
