# Supplementary material for: Green synthesis of silver nanoparticles from Brownlowia tersa leaf extract: multifaceted evaluation of antibacterial, antioxidant, cytotoxic, and anti-alzheimer potential
Source: PLoS One. 2025 Nov 4;20(11):e0335524. doi: 10.1371/journal.pone.0335524 (PMC12585095; doi:10.1371/journal.pone.0335524)
Supplement: Attachment — Submitted filename: Response_to_Reviewers_auresp_5.docx [file pone.0335524.s013.docx]

2^nd^ October 2025

To

Emily Chenette

Editor-in-Chief,

PLOS One,

Dear Editor,

Special gratitude to you for allowing us to resubmit a revised version of our manuscript entitled as **“Green Synthesis of Silver Nanoparticles from Brownlowia tersa Leaf Extract: Multifaceted Evaluation of Antibacterial, Antioxidant, Cytotoxic, and Anti-Alzheimer Potential”**, (Manuscript ID: PONE-D-25-15530R4) to the PLOS One as a research article. We also appreciate the time and efforts of you and your reviewers have given for this meaningful feedback, which we believe has greatly helped us to enhance the clarity of our article significantly. We have made changes based on your and the reviewers' suggestions, and the revised sections are highlighted in yellow color throughout the revised manuscript. We hope that our response, which is included below, will deal successfully with each reviewer's specific points. Please let us know if you have any additional questions and comments.

Therefore, we want to express our special gratitude to you for your consideration of this manuscript.

**Sincerely Yours,**

Md. Nazmul Hasan, PhD,

Professor,

Department of Genetic Engineering and Biotechnology,

Jashore University of Science and Technology,

Jashore-7408, Bangladesh.

E-mail: [mn.hasan@just.edu.bd](mailto:mn.hasan@just.edu.bd)

Google Scholar Profile: <https://scholar.google.com/citations?user=SP1f9JEAAAAJ&hl=en>

ResearchGate Profile: <https://www.researchgate.net/profile/Dr-Md-Nazmul-Hasan>

ORCID ID: <https://orcid.org/0000-0002-9122-1129>

**Journal Requirements:**

**Comments 1**). If the reviewer comments include a recommendation to cite specific previously published works, please review and evaluate these publications to determine whether they are relevant and should be cited. There is no requirement to cite these works unless the editor has indicated otherwise. 

**>>Response:** The reviewer did not suggest any additional references, and after reviewing the manuscript, I believe that the references currently included are appropriate and sufficiently support the study's findings and discussion. I have not added any new references since the existing ones cover the relevant literature comprehensively

Comments 2). Please review your reference list to ensure that it is complete and correct. If you have cited papers that have been retracted, please include the rationale for doing so in the manuscript text, or remove these references and replace them with relevant current references. Any changes to the reference list should be mentioned in the rebuttal letter that accompanies your revised manuscript. If you need to cite a retracted article, indicate the article’s retracted status in the References list and also include a citation and full reference for the retraction notice.

**>>Response:** I have carefully reviewed the reference list to ensure that all citations are complete and correct. I can confirm that none of the cited papers have been retracted. If any retracted articles had been cited, I would have followed the guidelines by including the rationale in the manuscript or replaced them with relevant current references.

No changes were made to the reference list, as all citations are up-to-date and appropriate. If any modifications were made, they will be mentioned in the rebuttal letter accompanying the revised manuscript.

**Responses of Editor Comments**

**Comments of Editor 1:**

Comment 1).  We have received the timely revised version, but the response regarding the mentioning of FT-IR ( specified wave number in discussion and spectra) and UV peaks (spectra) are still lacking.

**>>Response:** I rewrite again.

**Comments of Editor 1:**

Comment 2). The Fig 4 : the green highlighted number mentioning size are still unclear.

**>>Response:** I again increase resolution of these figure.

**Comments of Editor 1:**

Comment 3). ATCC code of microorganisms should be mentioned.

**>>Response:** The bacterial strain used in this study does not have an ATCC accession number. It is a laboratory-maintained isolate preserved in our departmental culture collection, and no standard culture collection code is available.

**Comments of Editor 1:**

Comment 4). Along with this, through out the article basic grammatical errors like (line 28: an LC₅₀ , line 29 Swiss albino, line 157: humane, line 293: Nutrient agar , line 254 : Artemia salina , line 391: p-value , (should be italic), abbreviations minutes, hours and spelling mistakes (gallic acid/ galic acid) are quite frequent and should be corrected.

**>>Response:** I have corrected it.

**Comments of Editor 1:**

Comment 5). Table 3 and figure 8 S.Aureus should be corrected.

**>>Response:** I have done it.

**Responses of Editor Comments**

**Comments of Editor 1:**

Comment 6).  Table 7 replace comma with a dot.
**>>Response:** I have done it

**Comments of Editor 1:**

Comment 7). Why supplementary figure S4, 5 and 6 with 2D and 3D images are represented separately?

**>>Response:** We represented Supplementary Figures S3, S4, S5, and S6 separately to provide both 2D and 3D visualizations of each protein–ligand complex. Since our study involves four different proteins with their respective ligands, presenting the 3D docking poses alongside the 2D interaction diagrams allows a clearer understanding of binding orientation, key interacting residues, and molecular contacts. This dual representation ensures a more comprehensive depiction of the binding interactions that could not be fully conveyed by a single format alone.

**Comments of Editor 1:**

Comment 8). The text fonts should be same throughout the article including figure and graphs.

**>>Response:** I have done it.

**Comments of Editor 1:**

Comment 9). As mentioned earlier, kindly go through the article to improve the sentences (section 2.1.4.3, 2.25.2, ) and quality of article so that it can meet the publishing quality of the journal.
**>>Response:** I have changed and rewritten according to your instructions.
